# Learning to read in first grade through a reading tutor that employs automatic speech recognition: The effects of implicit and explicit feedback

**DOI:** 10.1007/s10758-025-09860-8

**Published:** 2025-06-27

**Authors:** Yu Bai, Ferdy Hubers, Catia Cucchiarini, Roeland van Hout, Helmer Strik

**Affiliations:** 1 Centre for Language and Speech Technology (CLST), Radboud University, Nijmegen, The Netherlands; 2 Centre for Language Studies (CLS), Radboud University, Nijmegen, The Netherlands; 3 Donders Institute for Brain, Cognition and Behaviour, Radboud University, Nijmegen, The Netherlands

**Keywords:** Feedback, Reading tutor, Speech recognition, Decoding skills

## Abstract

Learning to read is an essential part of children’s overall development, and crucial in engaging them in our knowledge society. In this study, we investigated the effectiveness of two feedback conditions—explicit and implicit—on reading accuracy among first-grade pupils using our Automatic Speech Recognition (ASR) based Dutch reading tutor. Data were collected from 525 participants across 44 schools, with 263 pupils who received explicit feedback and 262 receiving implicit feedback. Mixed linear regression analyses revealed that both feedback conditions returned significant positive effects on reading accuracy in general, but, additionally, explicit feedback was more beneficial for accuracy gains in reading sentences. A trade-off between accuracy and reading speed was also observed, with improvements in accuracy often accompanied by slower reading speed. These findings suggest that explicit feedback may be more beneficial in early reading interventions.

## Introduction

Being able to read is a prerequisite to participate in our knowledge society. Developing good reading skills is essential in acquiring knowledge and for school success in general. Recent reports have shown declining levels of reading proficiency among Dutch children (Verdegaal, 2021). Decoding skills are crucial in triggering children’s reading development (Kendeou et al., 2009; Speece and Ritchey, 2005; Lonigan et al., 2008). Teaching reading, particularly decoding skills requires sustained and intensive practice (Castles et al., 2018; Clay, 1987; National Reading Panel, 2000) that encompasses both accurate and fluent reading. To develop good decoding skills children need to learn grapheme-phoneme relationships and to apply this knowledge while reading, preferably at considerable speed. Guided reading aloud is an important way of actively practicing decoding skills while receiving feedback on reading errors by a teacher (National Reading Panel, 2000). However, this type of practice is difficult to realize in large classroom settings, because not all children can read aloud simultaneously. As a consequence, teachers are not always able to provide each child with sufficient individual guidance.

In recent years, technology-assisted language instruction has been used as a supplementary tool in traditional classroom instruction (Meredith, 2015; Lippert et al., 2020; Besser et al., 2022). Among the technologies that have been employed to benefit language learning, Automatic Speech Recognition (ASR) has emerged as a very potential solution to partly compensate for the limitations of traditional classroom instruction. In the wake of advances in ASR technology, researchers have been looking at ways of applying ASR in educational reading software, in so-called reading tutors (RTs). An RT employs ASR to “listen” to children reading aloud and to identify possible errors and provide instantaneous individual feedback, thus offering additional opportunities for practicing reading skills. This type of development already led to commercial products such as the Reading Assistant (http://www.readingassistant.com/), IBM RC (https://www.ibm.com/ibm/responsibility/downloads/initiatives/ReadingCompanion.pdf), the ReadingBuddy (http://readingbuddysoftware.com/), and Soliloquy Learning’s Reading Tutor (http://www.soliloquylearning.com/). However, for many of these commercial systems little information is available about specific aspects of the system infrastructure and the effectiveness of the products. Most of the projects that implemented ASR in their RTs and tested the systems in experiments with children addressed reading in English: LISTEN (Mostow, 2016), Reading Companion (RC) (Grueneberg et al., 2007; Kantor et al., 2012), and the Colorado Literacy Tutor (CLT) (Hagen et al., 2003). One project that was aimed at developing ASR technology for supporting reading in Dutch addressed reading assessment, but eventually did not develop ASR for online practice (Duchateau et al., 2009; Cleuren, 2009).

In the LISTEN and RC projects one type of feedback was implemented and the effect of practice with the RT was examined in a pre- and posttest design (Mostow et al., 2013, 2003; Reeder et al., 2015; Grueneberg et al., 2007; Kantor et al., 2012). To our knowledge, no studies have investigated how different types of feedback in an RT affect children’s reading performance during practice. This type of research could, however, provide valuable insights into how feedback affects reading development, which in turn could be used to inform the development of more effective types of feedback.

The present study aims to evaluate the impact of implicit and explicit feedback on first-graders’ reading accuracy and speed using an ASR-based Dutch reading tutor. Specifically, we address the following questions: 1. To what extent do implicit and explicit feedback provided by the reading tutor affect reading accuracy? 2. How do different feedback conditions impact the trade-off between reading speed and accuracy?

This paper is organised as follows. In Section 1.1, we look into previous studies on RTs, focusing on the feedback implemented in the systems and their effectiveness. We discuss previous studies on corrective feedback in learning to read in the classroom, which provide useful information on how the feedback in an RT could be designed (Section 1.2). The current study and the research questions are presented in Section 2. In Section 3, we explain the methodology of this study, including the infrastructure of our RT system, the experimental setup, and data processing and analysis. The results are presented in Section 4. We discuss our findings in Section 5 and draw conclusions in Section 6.

### Reading tutors

As explained above, an automatic RT is here defined as an automatic system that employs ASR to analyse reading performance and provides online instantaneous corrective feedback to children while reading aloud. While several studies have employed ASR for the purpose of reading assessment, rather few systems have been developed and evaluated that are in line with this definition of RT. This is understandable considering that developing such systems requires considerable effort and speech resources that are not always available in sufficient quantity, high-quality content material and the cooperation with primary schools to be able to conduct experiments with sufficient numbers of children and to collect evaluations of the children’s performance at different stages of the experiments.

Research that employs ASR technology for the purpose of reading assessment (Madnani et al., 2019; Black et al., 2007; Bolaños et al., 2011; Bernstein et al., 2017; Balogh et al., 2012), is somewhat less problematic because in this case the ASR can be used offline and the evaluations can be conducted on already available speech corpora, rather than in online experiments. This is to say that ASR-based reading assessment systems employ ASR technology under different conditions, offline instead of online, and for a different purpose, obtaining an overall evaluation of reading skills, rather than detailed evaluations that form the basis for providing immediate feedback on individual items. An ASR-based reading assessment system can in any case be used to provide some form of performance feedback, for instance after a child has finished a number of exercises. The purpose of a reading assessment tool is to provide an overall evaluation of a child’s reading performance (such as overall reading accuracy and fluency) (Loukina et al., 2017, 2019; Madnani et al., 2019; Black et al., 2007; Bolaños et al., 2011; Bernstein et al., 2017; Balogh et al., 2012). Studies on reading assessment can be relevant for our research to determine which automatic measures can be employed to calculate reading accuracy and speed. However, since interactive feedback on reading errors is not implemented in reading assessment systems, these studies cannot provide information on how feedback influences children’s reading performance.

Reading assessment systems are basically different from automatic RTs. RTs should function as teachers who actively assist children to help them improve their reading skills, while reading assessment systems work as evaluation instruments. Research (Mostow, 2016; Kantor et al., 2012; Hagen et al., 2003; Li et al., 2008) has shown that software with ASR technology can be used as an RT which listens and provides instantaneous feedback to students. Although different types of assistance, for example, spoken feedback, graphical feedback, recast, encouragement, and highlighting, were implemented in some systems (Mostow and Aist, 1999; Kantor et al., 2012), the effectiveness of the various forms or types of feedback was not investigated. In previous studies, the rationale behind the choice of the feedback forms was either to: 1) to visualize the ASR results as much as possible, 2) to simulate what teachers do in the classroom. In both LISTEN and RC feedback was given immediately after children finished reading a sentence/phrase, explicitly in LISTEN and implicitly in RC (Kantor et al., 2012). In the study by Li et al. (2008), the system only provides help when users click for help. It appears that the effectiveness of different types of feedback on reading performance was not specifically addressed in the previous studies when the systems were developed.

Investigating the effects of different types of feedback in RTs is important because along with the performance of the ASR backend, the feedback from the RT may determine how much learners improve. Therefore, it is relevant to find out whether the effects of different feedback conditions vary and, if so, in what way they vary and how much. Moreover, in previous studies (Mostow et al., 2003, 2013; Reeder et al., 2015), the evaluation of the effectiveness of an RT, determined by measuring the improvement of the participants’ reading skills, was based on pre- and posttests after using the RT for a period of several months. This gives a rather global long-term view of gains in reading abilities, but more detailed analyses of how children’s reading performance is influenced by the assistance of an ASR-based RT during practice are currently lacking.

### Feedback in learning to read

The feedback generally provided by RTs and reading assessment tools falls in two categories: corrective feedback (CF) and performance feedback (PF). Some systems only provide CF or PF, some of them provide both. In this section, we focus on CF, which is necessary for children at an early stage of learning to read because they are sometimes unable to correct themselves. Most studies on the effects of feedback in learning to read have been conducted in traditional classroom situations. Corrective feedback (CF) has been proven to have a positive effect on oral reading by children, both with and without learning difficulties (Pany and McCoy, 1988; Perkins, 1988; Heubusch and Lloyd, 1998; Martin-Chang, 2017).

Studies on the effect of CF distinguish different types of CF based on several important characteristics (e.g., (Metcalfe et al., 2009; Swart et al., 2019; Watson et al., 2009; Cheatham et al., 2015; Netz and Fogel, 2019)): (1) timing: when the feedback is provided; (2) grain-size: whether the feedback is on sentence level, word level, syllable level, or phone level; (3) phonics-based vs. whole-word: whether feedback is provided by sounding out the whole word or phoneme-by-phoneme.

With respect to timing, Heubusch and Lloyd Heubusch and Lloyd (1998) suggested that feedback on oral reading should be provided immediately after the error occurs. This suggestion was based on their review of 24 studies focusing on correction techniques and correction timing and format for children with learning difficulties.

Studies focusing on the whole-word and phonics-based feedback distinction revealed that whole-word feedback is more effective than phonics-based feedback with regard to reading accuracy and reading rate (Spaai et al., 1991; Watson et al., 2009). Heubusch and Lloyd (1998) suggested that this depends on the reading goals. In some cases, it might be more effective to use word supply feedback (providing the correct words following an error), while in other cases phonetic emphasis feedback (providing one sound at a time) is more appropriate.

In addition to the three characteristics presented above, studies on the effect of CF in the context of second language (L2) learning have identified another dimension along which feedback forms can be distinguished: input-providing vs output-pushing. In input-providing feedback, such as recast and explicit correction (Lyster, 2004; Ellis, 2006), the correct form is provided, while in output-pushing feedback, which includes elicitation, meta-linguistic feedback, clarification requests, and repetition (Lyster, 2004) the correct forms are withhold and learners are pushed to self-correct based on their existing knowledge (Ellis, 2009).

These types of feedback have been mainly investigated in the context of children’s dual language learning and L2 development. By studying teachers’ feedback strategies for dual language children, Cheatham Cheatham et al. (2015) observed that both implicit and explicit input-providing feedback and output-pushing feedback are used in the classroom. Netz and Fogel Netz and Fogel (2019) compared the effect of input-providing and output-pushing corrective feedback on students’ responses and found that students managed to correct themselves more often when they received input-providing feedback (recasts and explicit corrections), than when they received output-pushing feedback (prompts). For prompts, pupils needed more attempts to reach the correct answers, but qualitative analyses revealed that output-pushing was more conducive to long-term gains.

Although these specific forms of CF have not been studied in connection with learning to read, it seems useful to find out to what extent they are effective in this context, especially because both input-providing and output-pushing feedback can be relatively easily implemented in an RT to assist pupils in correcting reading errors.

The feedback on children’s reading aloud provided by teachers in the classroom is usually immediate and implicit. Teachers interrupt when the pupil makes a reading error immediately or after the pupil finishes the phrase or sentence. However, teachers tend not to tell pupils explicitly that they were wrong in order to create a respectful and encouraging learning environment. We know, however, from research on Computer Assisted Language Learning that these possibly negative effects of explicit corrective feedback can be attenuated in a context in which there is no risk of losing face like a one-on-one interaction with an RT or CALL system (Bodnar, 2016). Given that most previous studies on corrective feedback have almost exclusively focused on classroom instructions, it is interesting now to examine the effects of different forms of CF in reading assistant software.

## Current study

In the present paper, we configured a Dutch RT in different ways to implement four types of feedback: implicit-delayed, explicit-immediate feedback, and input-providing and output-pushing feedback. We constructed two feedback conditions, explicit versus implicit. The research question we addressed is:

*Do the explicit and implicit feedback conditions differ in the extent to which they lead to increased reading accuracy?*

We developed an RT equipped with logging capabilities for Dutch first graders (Bai et al., 2020; Bai et al., 2021b) that uses ASR to compare the words read aloud by children to the prompts displayed on the screen and gives feedback based on this evaluation. The initial version of the Dutch RT was developed in YEAR XXX after consultations with teachers and reading experts. We employed ASR technology developed by the company XXX and reading exercises designed by XXX Publishers (Bai et al., 2020). We then followed the process of “Development - Experiment - Analysis”. First, a pilot experiment with several first grade students was conducted at a XXX primary school. The first official version was used in the first experiment by pupils from six schools at home. With the log data, we were able to improve the system according to the analyses of the log files collected.

We implemented two feedback conditions in the RT system. Feedback was given on the word level, both in the implicit and explicit experimental conditions. In the explicit condition, immediate feedback was given on the word level when a mistake was made. In the implicit condition, feedback was delayed, meaning that students received feedback after finishing the exercise. In both conditions, students received output-pushing feedback. If they did not manage to self-correct, input-providing feedback was given. The tutor system gave the students multiple chances to reach the correct form.

One of the important features of our RT is its logging capabilities. Every time a child finishes an exercise, information including student ID, prompt, and time when the exercise was finished is sent to the server along with the ASR results of each word (Bai et al., 2020; Bai et al., 2021b). With the log data, it is possible to keep track of the children’s reading progress in accuracy and speed during practice. Moreover, we can even look into how their reading accuracy and speed changed for the same word at different attempts.

## Methodology

### Dutch automatic reading tutor

#### Reading materials

The reading materials incorporated in the software were taken from the reading method ’XXX’ designed by XXX Publishers (Mommers et al., 1990), which is used by the majority of primary schools in the COUNTRY to teach pupils to read texts of increasing difficulty levels (Mommers et al., 1990). More specifically, we used the materials from the practice book XXX (chapters 9 and 10), which first graders use to practice their decoding skills.

Two types of exercises are included: accuracy exercises and fluency exercises. The accuracy exercises aim at improving pupils’ reading accuracy of individual words and sentences and are specifically used to practice newly learned word types. In contrast, the fluency exercises aim at improving both pupils’ reading accuracy and reading speed and are used to practice word types pupils are already familiar with. Both exercise types were implemented in the software. However, the crucial difference between the exercises in the software and the exercises in the practice book is that the software provided corrective feedback during practice, while pupils usually have to complete the exercises in the practice book independently, often without individual guidance and feedback. In the current paper, we focus on the accuracy exercises only. A description and analysis of the fluency exercises are reported in Bai et al. (2022).

#### Front-end: exercises and feedback

The accuracy exercises in the system came in two feedback conditions we refer to as ’explicit feedback’ and ’implicit feedback’ respectively, although these conditions differ in more respects than only the explicit/implicit distinction, as described above in Sect. 2. Table 1 gives a detailed overview of the characteristics of these conditions.

**Table 1:** An overview of the features of the two feedback conditions

Feedback condition	Feedback cycle	Timing	Explicitness	Input/output
1. Explicit feedback	1	Immediate	Explicit	Output-pushing
	2	Immediate	Explicit	Input-providing
2. Implicit feedback	1	Delayed	Implicit	Output-pushing
	2	Delayed	Implicit	Input-providing

**Fig. 1:**
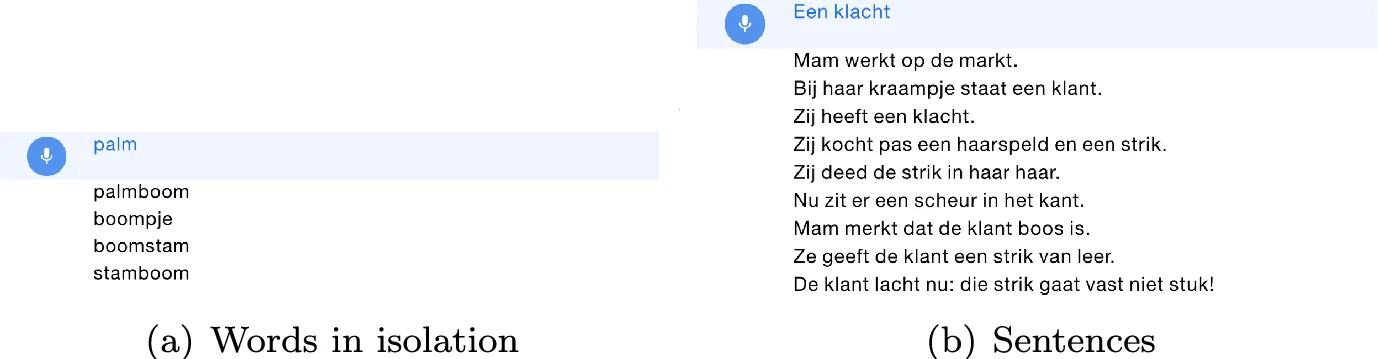
The first attempt of reading (a) a list of isolated words and (b) a short story

The way pupils read a word or sentence for the first time (first attempt) was similar in both feedback conditions. See Fig. 1. Pupils always started by clicking the record button in front of the prompt (either a single word, Fig. 1a or a sentence, Fig. 1b), and subsequently read the item aloud. Then the software analyzed whether it was read correctly or not. If it was read incorrectly, pupils had to read the prompt again either immediately (in the explicit feedback condition) or later on in the exercise (in the implicit feedback condition). Pupils maximally got three attempts to read each word or sentence correctly. In total, pupils could receive feedback twice: in between the first and second attempt (the first feedback cycle), and in between the second and third attempt (the second feedback cycle). In both feedback conditions, feedback was output-pushing in the first feedback cycle, meaning that pupils had to try again without hearing the correct realisation of the prompt. In the second feedback cycle, feedback was always input-providing: pupils were first presented with the correct form of the word or sentence, after which they had to read it again themselves.

In the explicit feedback condition, pupils received feedback immediately after their attempt. If they read the word correctly (in case of reading isolated words), a green check mark appeared in front of it. If they read the word incorrectly at their first attempt, the word was underlined in orange and pupils were asked to read the word again (output-pushing). If the answer was correct at the second attempt, a green check mark appeared in front of the word and the record button moved to the next word. If the second attempt was still incorrect, the software played the correct word form (input-providing) and then asked the pupil to try again (third attempt). The pupil could re-listen to the correct speech by clicking the play button next to the word. If the answer was correct, a green check mark appeared in front of the word and the record button moved to the next item. If not, the orange underlining remained on the current item and the record button went to the next word. The procedure above was very similar for reading sentences. Words that were read incorrectly were highlighted using the orange underlining. If all words were read correctly, a green check mark appeared in front of the sentence. Before the third attempt, the entire sentence was played if it still contained incorrectly read words at the second attempt.

In the implicit feedback condition, feedback was delayed and implicit. Feedback was not provided immediately after the pupil finished reading a word or sentence, but only after all words or sentences of that exercise had been read. Moreover, feedback was implicit rather than explicit, meaning that incorrectly read words were not underlined and correctly read words were not accompanied by a green check mark. Instead, pupils were asked to read a subset of the words or sentences again (the incorrect ones), without explicitly indicating that these items had been read incorrectly before. So, after pupils read all words (in case of reading isolated words) for the first time (first attempt), pupils were presented with the words that were read incorrectly at the first attempt. These words appeared in a different order and pupils were asked to read these words again (output-pushing) in a similar one by one fashion, clicking the record button each time. If all words were read correctly at the first attempt, pupils continued to a new set of items. Words that were also read incorrectly at the second attempt, were subsequently presented on a new screen, again in a different order. Now the software played the correct word forms first (input-providing) after which the words were shuffled and pupils were instructed to read the words again. This procedure was very similar for reading sentences. However, after the first and second attempt, also the correct sentences were presented on the screen (and not only the incorrect ones), because all sentences together formed a story and presenting only the incorrectly read sentences would disrupt the story. At the second attempt, the computer and the pupils read the story together: sentences that were read correctly before were now played by the software, while the incorrect sentences were read by the pupils without hearing the correct form first (output-pushing). At the third attempt, all sentences (correct and incorrect ones) were played by the software and only the incorrect ones were read by the pupils.

#### Back-end: ASR technology, system scoring and logging

The reading tutor consisted of a front-end (the user interface, described in 3.1.2) and back-end with the ASR. For the back-end, we made use of the COMPANYXXX ASR. On the front-end words were displayed on the screen. If a pupil clicked the record button, the recording started and the audio file was sent to the back-end. Subsequently, the ASR analysed the recorded utterance, and calculated probability scores at the phone level, which were expressed in numbers ranging from 0 to 100. The probability scores expressed the extent to which the phones in the recorded utterance were similar to the phones in the prompt, with higher scores indicating increasing similarity. The probability score at the word level was subsequently computed by taking the average probability score of all phones. The acoustic model of the COMPANY XXX ASR was trained on Dutch adult speech.

**Fig. 2:**
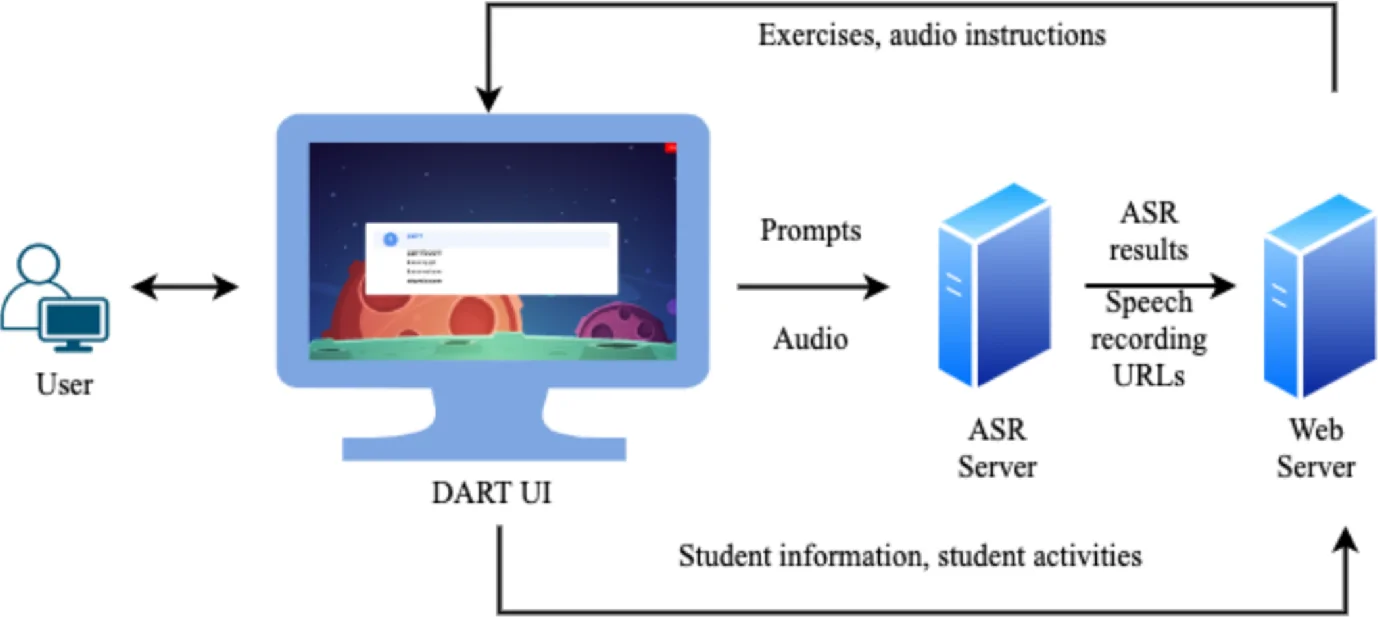
The infrastructure of the Dutch Reading Tutor

For each recording, a log file was generated that contained detailed information, such as the onset and offset of a word, the phone probability scores and the probability scores at word level, the system judgement (correct/incorrect), and the attempt number. This data is later used for evaluating both reading accuracy and fluency. Figure 2 illustrates the infrastructure of the reading tutor system.

Based on the word probability score, the software determined whether a word was read correctly or not. If the probability score of a word equalled or exceeded our threshold of 73, the word was considered to be read correctly, while words with a probability score lower than 73 were treated as incorrect. This threshold was determined based on a previous study in which we compared the system judgements to human annotations and concluded that this was the optimal threshold (Bai et al., 2021a). Reading accuracy difference scores were determined by subtracting the word probability score of the first attempt from that of the second attempt. A similar approach was used to calculate reading speed, where the number of graphemes per second from the first attempt was subtracted from the number of graphemes per second on the second attempt.

### Experimental design

#### Participants

In total, 525 Dutch first graders (age between 6 and 7 years old) from 44 primary schools worked with the software at school on their school computers. Within each class, pupils were randomly assigned to one of the two feedback conditions. In total, 263 pupils worked with the system in the explicit feedback condition, and 262 pupils were assigned to the implicit feedback condition.

#### Procedure

The pupils worked with the software in one of the two feedback conditions for a period of six weeks (chapters 9 and 10 of ’READING METHOD XXX’). During the six weeks of practice, teachers were instructed to let pupils work with the software minimally twice a week, preferably in a quiet environment (e.g., separate classroom). While most pupils did manage to use the software twice a week, there was not always a quiet room available.

#### Data processing and analysis

The log files generated by the software (raw data) were merged into one data file using Python 3 (Van Rossum and Drake, 2009). Additional data processing and statistical analyses were performed using R, version 4.1.3 (R Core Team, 2022) and the R-packages *Tidyverse* (Wickham et al., 2019), *lme4* (Bates et al., 2015), *sjPlot* (Lüdecke, 2022), and *MuMIn* (Barton, 2022).

To answer our research question, we first calculated difference scores between two consecutive attempts at reading the same word by the same pupil in the reading accuracy exercises. The reading accuracy difference scores were calculated by subtracting the word-level probability score of a word at the first attempt from the word-level probability score of the same word at the second attempt. This way, a positive difference score indicates an improvement in terms of reading accuracy, while a negative difference score indicates a deterioration.

After having calculated the difference scores, we carried out a linear mixed effects regression analysis in which we used the accuracy difference scores as the dependent variable. Following recommendations from Heck, Thomas, and Tabata Heck et al. (2014), variables were selected based on their theoretical relevance to the study’s research questions. Feedback condition, feedback cycle, word length, word context (reading isolated words (word lists) versus words in sentences, related to two reading taks) were treated as fixed effects, while individual differences among pupils (e.g., baseline reading ability), words, and schools were modeled as random effects. We started off with a model that included random intercepts for words, pupils and schools to account for the nesting in our data. In addition, we included feedback condition (explicit vs. implicit feedback), feedback cycle (1, output-pushing vs. 2, input-providing) and their interaction as fixed effects, because they were crucial to answering our research question. Subsequently, we added other predictors (random and fixed factors) one by one to the model based on theory, and examined whether the model fit improved based on Likelihood Ratio Tests. If this was not the case, the predictor was not included in the model. We used the package *lmerTest* (Kuznetsova et al., 2017) to calculate *p* values for the predictors in the model, and R-squared as a measure of the model fit.

## Results

Table 2 presents descriptive statistics for the reading accuracy difference scores of pupils by feedback condition (explicit vs. implicit) and by feedback cycle (the first cycle, output-pushing feedback, or the second cycle, input-providing feedback). It appears that all four mean difference scores were positive, with small confidence intervals, and far from zero. This indicates that both feedback conditions (explicit and implicit) led to improved reading accuracy in pupils in both feedback cycles. The average reading accuracy gains are higher for explicit than for implicit feedback, and within explicit feedback the average gains are higher in the second cycle (than the first cycle).

**Fig. 3:**
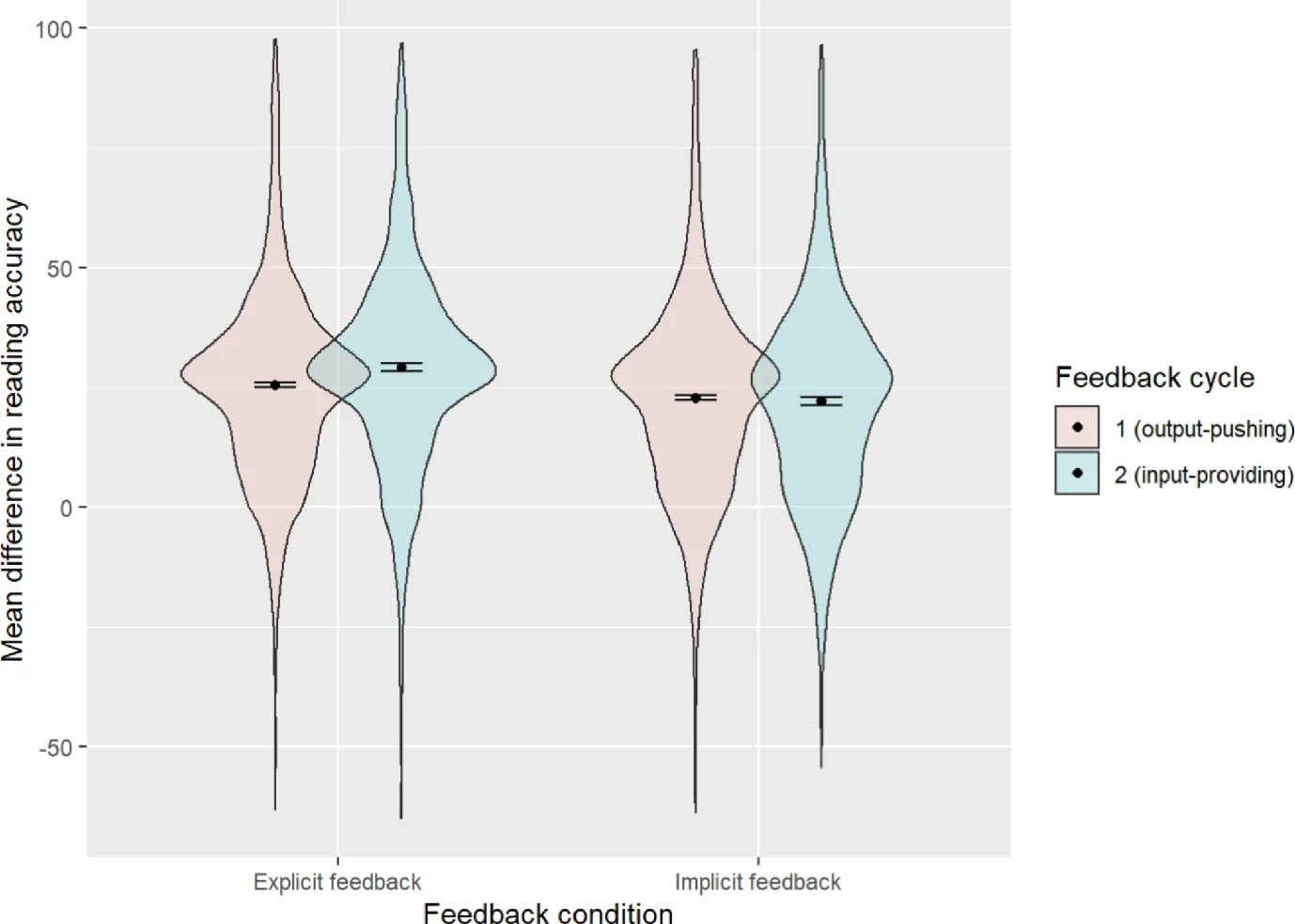
Violin plots with the mean reading accuracy difference scores by feedback condition and feedback cycle. Error bars represent 95% confidence intervals

In addition to the mean difference scores and confidence intervals, the violin plots in Fig. 3 provide additional information about the distribution of the scores. They show that only a minority of the scores were negative and that the four distributions involved have a similar shape with a wide range.

**Table 2:** Average reading accuracy difference scores, their standard deviations (SD) and confidence intervals (CI) by feedback condition and feedback cycle

Feedback condition	Feedback cycle	Mean	SD	CI
Explicit feedback	1 (output-pushing)	25.51	19.48	25.05;25.97
	2 (input-providing)	29.24	20.49	28.42;30.06
Implicit feedback	1 (output-pushing)	22.76	20.00	22.27;23.26
	2 (input-providing)	22.02	20.82	21.18;22.87

To examine the effects in Table 2 properly and to test the effectiveness of feedback, we had to take into account all relevant variables present in our experimental design using linear mixed effects regression analysis. Our final regression model contained the following fixed effects: feedback condition, feedback cycle, word length, word context, a three-way interaction between feedback condition, feedback cycle, and word context and their two-way interactions. Words (random intercept only), pupils (random intercept and random slope of feedback cycle) and schools (random intercept only) were included as random effects. All categorical predictors were treatment coded, the reference categories being ’explicit’ for feedback condition, ’cycle 1 (output-pushing)’ for feedback cycle, and ’words in isolation’ for word context. The outcomes of the regression model are presented in Table 3.

**Table 3:** Final regression model of reading accuracy difference scores

Fixed effects		Beta	SE	*t*	
Intercept		27.99	1.00	27.85	***
Feedback condition (explicit vs. implicit)		0.70	0.68	1.03	
Feedback cycle (1 vs. 2)		3.80	0.99	3.83	***
Word context (word in isolation vs. word in sentence)		5.10	0.58	8.87	***
Word length		-1.25	0.14	-8.76	***
Feedback condition x Feedback cycle		-0.62	1.42	-0.44	
Feedback condition x Word context		-4.05	0.70	-5.76	***
Feedback cycle x Word context		-0.47	1.08	-0.43	
Feedback condition x Feedback cycle x Word context		-3.31	1.54	-2.14	*
Random effects		Var.	SD	Corr.	
Word	Intercept	16.66	4.08		
Pupil	Intercept	13.09	3.62		
Pupil	Feedback cycle	14.37	3.79	-0.33	
School	Intercept	2.26	1.50		
Residual		333.58	18.26		

* *p* <.05; ** *p* <.01; *** *p* <.001; marginal $$R^2$$ =.039, conditional $$R^2$$ =.127

The effect of word length is rather straightforward, as it did not interact with any of the other variables. The longer the word, the less the improvement in reading accuracy (β = -1.25, *p* <.001). This effect was present in all experimental conditions. We observed a negative correlation (-0.33) for the feedback cycle in the random effects. This negative value suggests that as the number of feedback cycles increased, individual reading performance tended to decrease in variability. In other words, students became more consistent in their reading accuracy as they received more cycles of feedback. For the interaction terms, the analysis revealed non-significant results for both Feedback condition x Feedback cycle and Feedback condition x Word context. This indicates that the relationship between feedback type (implicit vs. explicit) and reading performance did not significantly change depending on the feedback cycle or the context of the words. These findings suggest that the effect of feedback condition remained stable across different cycles and word contexts.

**Fig. 4:**
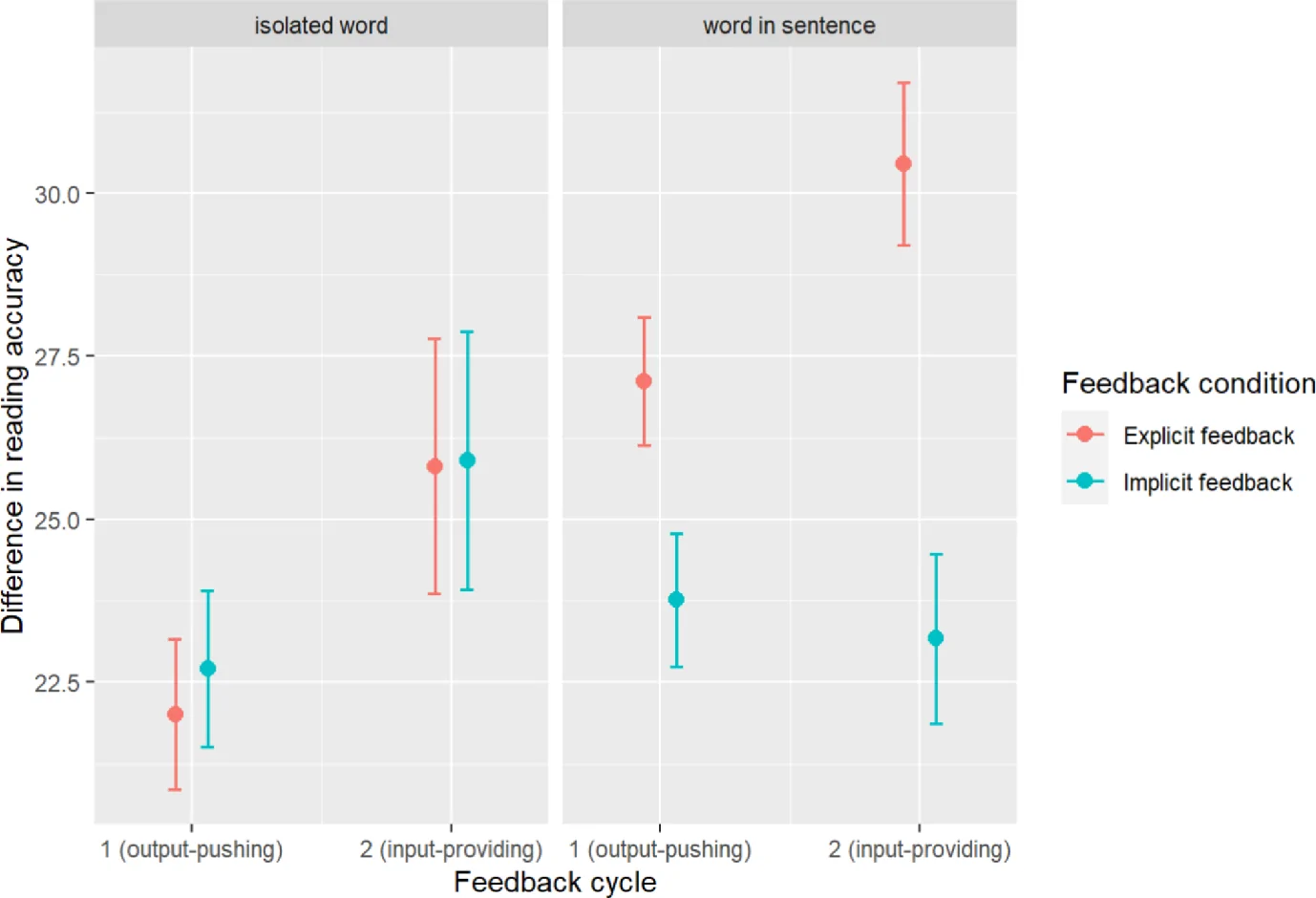
Reading accuracy difference scores (model estimates) by Feedback condition (explicit vs. implicit) and Feedback Cycle (1 vs. 2)

To evaluate the other effects we had to interpret the three-way interaction between feedback condition, feedback cycle, and word context. This interaction is visualized in Fig. 4. In this figure, the distinction in word context is the main distinction. In order to properly interpret the three-way interaction, we performed the same regression analyses for each word context separately, for isolated words and for words in sentences.

The results of the analysis for isolated words are presented in Table 4. In line with the left panel in Fig. 4, a positive effect of feedback cycle was found: Feedback cycle 2 (input-providing feedback) led to a significantly larger improvement in terms of reading accuracy than feedback cycle 1 (output-pushing feedback) (Explicit: β = 3.15, *p* =.001, Implicit: β = 2.35, *p* =.011). This effect was similar in both feedback conditions, as the interaction effect between Feedback condition and Feedback cycle was not significant (β = -0.80, *p* =.532). Moreover, implicit and explicit feedback did not significantly differ from each other at feedback cycle 1 (β = 0.44, *p* =.433) and feedback cycle 2 (β = -0.37, *p* =.740).

**Table 4:** Regression model of reading accuracy difference scores for isolated words only

Fixed effects		Beta	SE	*t*	
Intercept		22.88	1.18	19.35	***
Feedback condition (explicit vs. implicit)		0.44	0.56	0.79	
Feedback cycle (1 vs. 2)		3.15	0.90	3.50	***
Word length		-0.40	0.19	-2.15	*
Feedback condition x Feedback cycle		-0.80	1.28	-0.63	
Random effects		Var.	SD	Corr.	
Word	Intercept	14.43	3.80		
Pupil	Intercept	9.56	3.09		
Pupil	Feedback cycle	40.85	6.39	-0.78	
School	Intercept	0.10	0.32		
Residual		202.71	14.24		

* *p* <.05; ** *p* <.01; *** *p* <.001; marginal $$R^2$$ =.007, conditional $$R^2$$ =.120

The results for words in sentences are far more complex (see Table 5), as we did find a significant interaction between feedback condition and feedback cycle here (β = -3.96, *p* <.001) (see also right panel of Fig. 4). In both feedback cycles, explicit feedback was more successful than implicit feedback (Cycle 1: β = -3.42, *p* <.001, Cycle 2: β = -7.38, *p* <.001). However, the second feedback cycle (with input-providing feedback) led to a significantly larger improvement in reading accuracy compared to the first feedback cycle (with output-pushing feedback) in the explicit feedback condition only (β = 3.46, *p* <.001), while no such effect was observed in the implicit feedback condition (β = -0.50, *p* =.451).

**Table 5:** Regression model of reading accuracy difference scores for words in sentences only

Fixed effects		Beta	SE	*t*	
Intercept		35.62	1.07	33.35	***
Feedback condition (explicit vs. implicit)		-3.42	0.61	-5.61	***
Feedback cycle (1 vs. 2)		3.46	0.63	5.52	***
Word length		-1.75	0.19	-9.21	***
Feedback condition x Feedback cycle		-3.96	0.91	-4.36	***
Random effects		Var.	SD	Corr.	
Word	Intercept	17.24	4.15		
Pupil	Intercept	17.85	4.22		
Pupil	Feedback cycle	15.22	3.90	-0.39	
School	Intercept	3.60	1.90		
Residual		381.16	19.52		

* *p* <.05; ** *p* <.01; *** *p* <.001; marginal $$R^2$$ =.037, conditional $$R^2$$ =.127

## Discussion

The use of ASR-based Reading Tutors (RT) in this study builds on existing literature that highlights the effectiveness of automated tutoring systems in supporting early literacy. RT systems, particularly those that use ASR, provide students with individualized, real-time feedback that is difficult to achieve in large classrooms where teachers may not have the resources to provide personalized instruction to every student (Mostow and Aist, 2001; McCandliss et al., 2003). Research has demonstrated that these systems can significantly improve both reading fluency and accuracy, particularly when integrated with adaptive feedback mechanisms (Chiang and Jacobs, 2010). The ability of the ASR system to analyze phoneme-level data allowed the Reading Tutor in this study to provide targeted feedback, which likely contributed to the improvements in reading accuracy observed.

In our study, reading accuracy improvement was examined immediately after feedback was provided. We found that all parts in our experimental set-up (feedback condition by feedback cycle by reading task) led to reading accuracy gains in the accuracy exercises during practice. These results are in line with those of previous research in which longer term effects were observed, as accuracy improvements were seen after children had been using the RT for several months (Mostow et al., 2003, 2013; Reeder et al., 2015).

In addition, our results also showed that the magnitude of the accuracy gains was different for the two feedback conditions, depending on feedback cycle and reading task (isolated words vs. sentences). For reading isolated words, implicit and explicit feedback had similar effects on reading accuracy, while input-providing feedback (in cycle 2) had a stronger positive effect om accuracy than output-pushing feedback (in cycle 1). In other words, when reading isolated words, children’s accuracy gains were larger after input-providing feedback than after output-pushing feedback. In reading sentences, the two feedback conditions were clearly different. The explicit feedback condition returned higher accuracy effects in both cycles; and input-providing feedback (in cycle 2) led to larger accuracy gains than output-pushing feedback (in cycle 1). In the implicit feedback condition, the two cycles had a similar positive effect. The advantages of input-providing feedback is in line with studies in the field of second language learning (Netz and Fogel, 2019). These findings also align with previous research that demonstrates the benefits of immediate corrective feedback, especially in early literacy development. Immediate feedback helps learners quickly identify and correct errors, which has been shown to accelerate progress in reading skills (Van der Kleij et al., 2015; Hattie and Timperley, 2007). Conversely, the implicit feedback condition, which allowed students to self-correct, resulted in more gradual improvements. This finding is consistent with research suggesting that implicit feedback encourages students to engage in deeper cognitive processes, such as reflection and self-regulation. Studies on implicit feedback in learning environments indicate that delayed feedback can promote better long-term retention, as learners must process and correct errors independently (Butler and Winne, 1995; Nicol and Macfarlane-Dick, 2006).

In our paper that focused on the fluency exercises (Bai et al., 2022), we found that explicit feedback led to larger accuracy gains both in reading word lists and reading stories (two related but different reading tasks). Combined with the results in this paper, it seems that explicit feedback is beneficial to children’s reading accuracy especially when reading sentences, word lists, and stories. The fact that we did not find a difference between the explicit and implicit feedback condition when reading isolated words may be due to the system’s performance in evaluating isolated words. In Bai et al. (2021), we showed that the degree of agreement between the teachers and the system in detecting reading errors was lower in isolated words than in sentences, word lists and stories.

We also noticed that the largest decrease in speed occurred after explicit feedback in sentences (See Appendix). This suggests that there is a trade-off between reading accuracy and speed. Pupils had to slow down to improve reading accuracy. This finding is also in accordance with the results reported in (Bai et al., 2022).

The results also demonstrate that there is less reading accuracy improvement for longer words, which is in accordance with the finding that in Dutch word-decoding, children are most efficient at reading words with CVC patterns and polysyllabic words are more challenging (Verhoeven and Leeuwe, 2009).

## Conclusion

In our study, we implemented two feedback conditions in our Dutch RT system. In the explicit feedback condition, children received explicit and immediate feedback, while in the implicit feedback condition, implicit and delayed feedback was given. We found that all parts in our experimental set-up (feedback condition by feedback cycle by reading task) led to reading accuracy gains, but that the magnitude of these gains varied in the different experimental parts.

For isolated words, larger improvements were seen after input-providing than after output-pushing feedback, while no differences were observed between explicit-immediate and implicit-delayed feedback. For sentences, we did find such a difference, as explicit-immediate feedback resulted in larger improvements in reading accuracy than implicit-delayed feedback. Moreover, input-providing feedback was more effective than output-pushing feedback, but only in the explicit feedback condition.

To summarize, for isolated words the advantage of input-providing feedback emerged in both the explicit and the implicit conditions. For sentences, the leverage of input-providing feedback was only revealed in the explicit feedback condition. In addition, we saw less accuracy gains for longer words after all conditions of feedback.

The insights gained in this study contribute to our understanding of the role of practice and feedback in ASR-based RTs in supporting reading development. In previous studies (Mostow et al., 2003, 2013; Reeder et al., 2015; Grueneberg et al., 2007; Kantor et al., 2012) on ASR-based RTs, only one condition of feedback had been implemented into the systems and the evaluations had not investigated the effects of feedback during practice. We implemented different conditions of feedback to study possible improvements during practice. The results of this study indicate how children’s reading accuracy is influenced by feedback conditions in the system, which can inform the development of future RTs in terms of feedback configuration. More importantly, the broad implication of the present research is that the traditional classroom reading instruction practice can be complemented by educational software for reading aloud, in which the interaction between teacher and students is supported by ASR technology.

## Figures and Tables

**Fig. 5:**
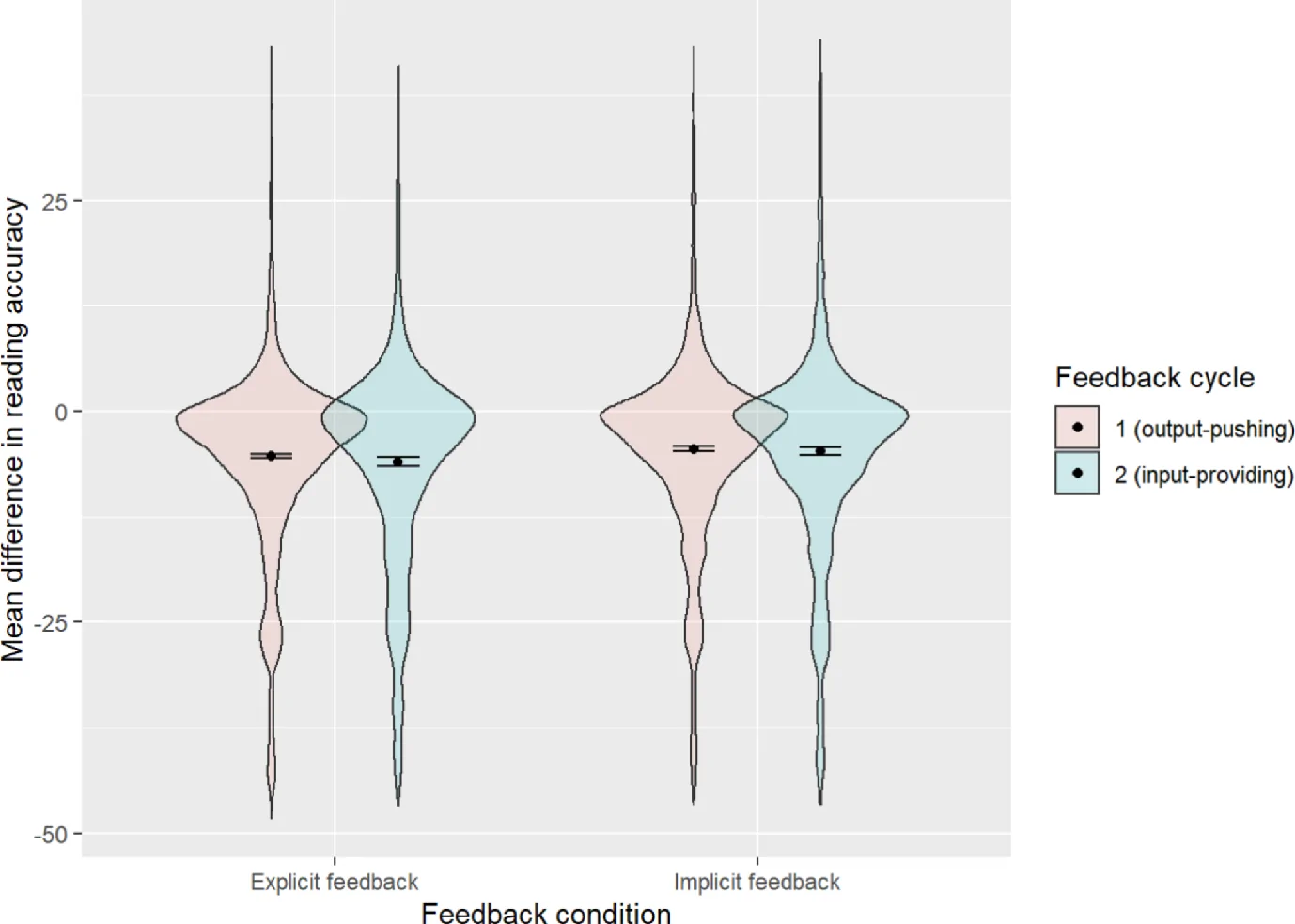
Violin plot with the mean reading speed difference scores by feedback condition and feedback cycle. Error bars represent 95% confidence intervals

**Fig. 6:**
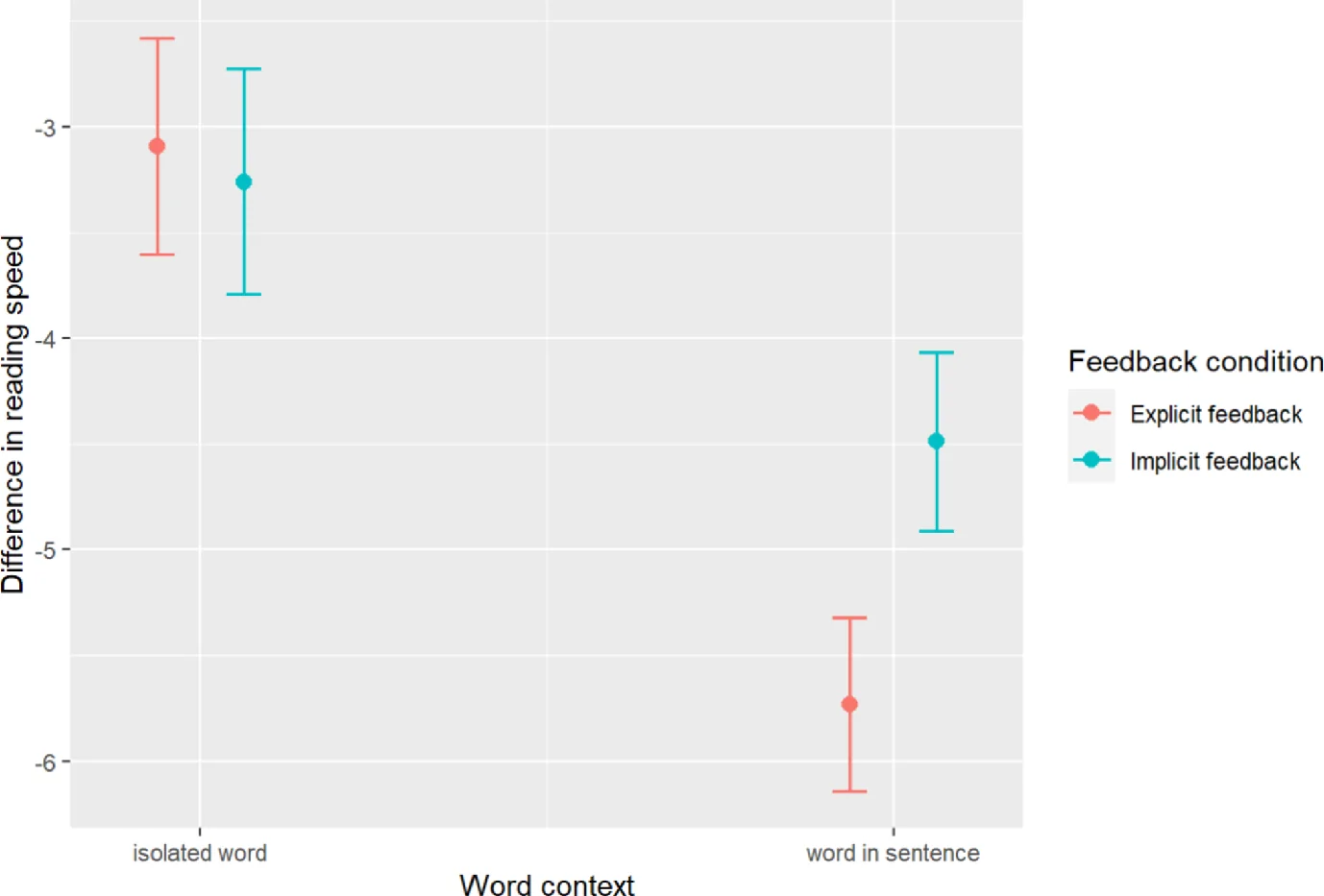
Reading speed difference scores (model estimates) by Feedback condition (explicit vs. implicit) and Reading context (isolated word vs. word in sentence)

**Fig. 7:**
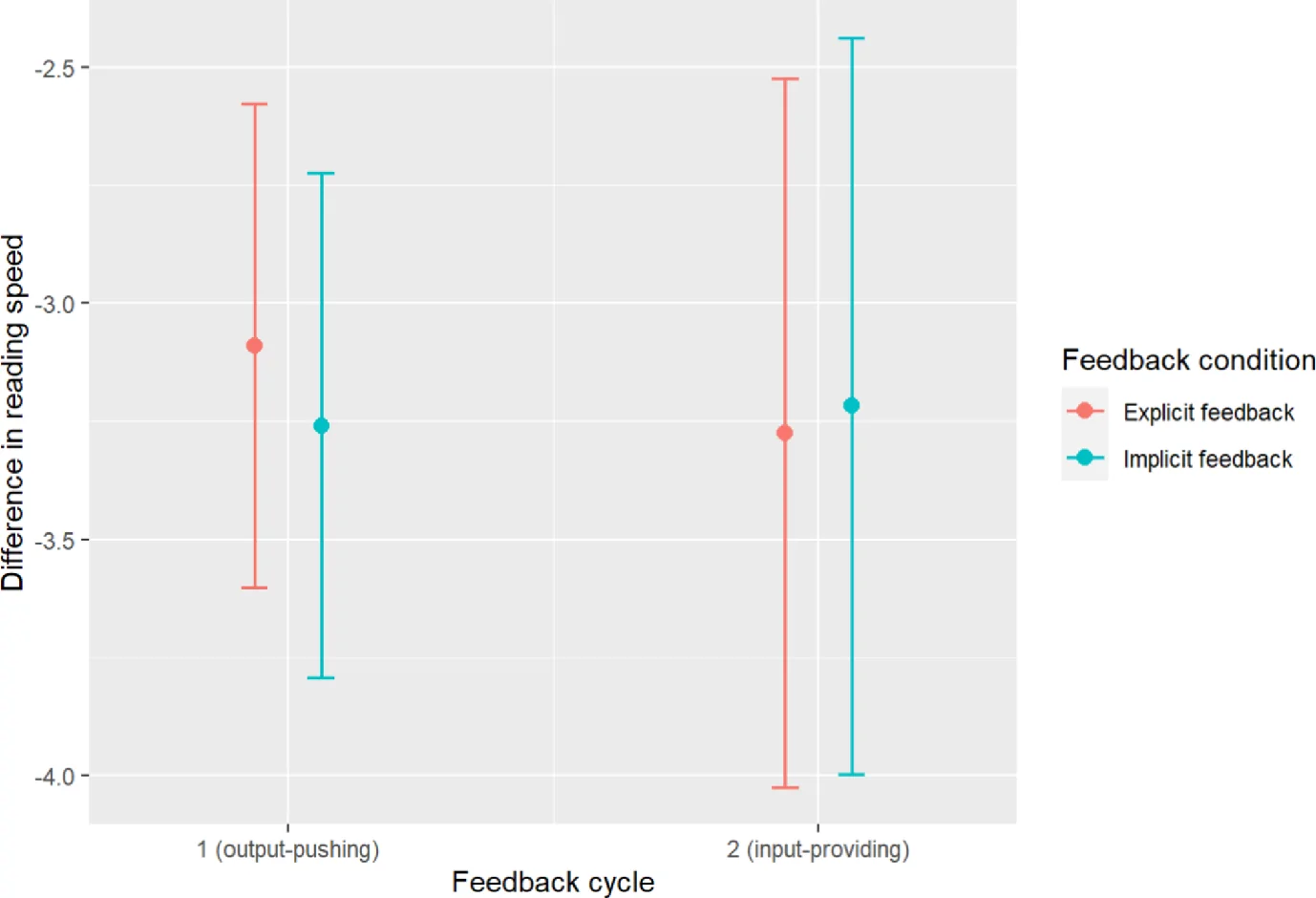
Reading speed difference scores (model estimates) by Feedback condition (explicit vs. implicit) and Feedback cycle (1 vs. 2)

## Data Availability

The datasets generated during and/or analyzed during the current study are available from the corresponding author on reasonable request.

## Appendix

### Reading speed analysis

The descriptive statistics for the reading speed difference scores of pupils by feedback condition (explicit vs. implicit) and by feedback cycle (first cycle, output-pushing feedback, vs. second cycle, input-providing feedback) are presented in Table 6. The difference scores in all four conditions are negative and suggest that pupils slow down (read slower) at the current attempt as compared to the previous attempt. This slowdown seems to be somewhat larger in the explicit feedback condition, than in the implicit feedback condition. Figure 5 presents the distribution of the difference scores by Feedback condition and feedback cycle. It shows that, although pupils speed up in some cases (positive difference scores), most of the time they slow down.

**Table 6:** Average reading speed difference score between two attempts, their standard deviations (SD) and confidence intervals (CI) by feedback condition and feedback cycle

Feedback condition	Feedback cycle	Mean	SD	CI
Explicit feedback	1 (output-pushing)	-5.36	10.58	-5.61;-5.12
	2 (input-providing)	-5.97	11.99	-6.45;-5.49
Implicit feedback	1 (output-pushing)	-4.44	10.67	-4.71;-4.18
	2 (input-providing)	-4.72	11.50	-5.19;-4.26

A linear mixed effects regression analysis was conducted to actually test whether the observed differences in Table 6 are statistically significant, while taking into account other relevant variables. The final regression model contained the following fixed effects: feedback condition, feedback cycle, word length, and a two-way interaction between feedback condition and feedback cycle and between feedback condition and word context. We included Words (random intercept only), and Pupils (random intercept and random slope of Feedback condition) as random effects. School could not be included as a random effect, because the variation between schools was too limited. All categorical predictors were treatment coded and the reference categories were ’explicit’ (Feedback condition), ’cycle 1 (output-pushing)’ (Feedback cycle), and ’word in isolation’ (Word context). The regression model is presented in Table 7.

**Table 7:** Final regression model of reading speed difference scores for the accuracy exercises

Fixed effects		Beta	SE	*t*	
Intercept		-5.30	0.49	-10.73	***
Feedback condition (explicit vs. implicit)		-0.17	0.34	-0.52	
Feedback cycle (1 vs. 2)		-0.18	0.35	-0.53	
Word context (word in isolation vs. word in sentence)		-2.64	0.29	-9.10	***
Word length		0.46	0.07	6.36	***
Feedback condition x Feedback cycle		0.22	0.50	0.45	
Feedback condition x Word context		1.41	0.35	4.04	***
**Random effects**		**Var.**	**SD**	**Corr.**	
Word	Intercept	3.67	1.92		
Pupil	Intercept	1.62	1.27		
Pupil	Feedback cycle	10.19	3.19	-0.49	
Residual		104.39	10.22		

* *p* <.05; ** *p* <.01; *** *p* <.001; marginal $$R^2$$ =.022, conditional $$R^2$$ =.083

The regression model shows a main effect of word length, as it is not in interaction with the other variables in the analysis. This positive effect indicates that the longer the word, the less pupils slowed down after feedback (β = 0.46, *p* <.001). The interaction effect between feedback condition and word context was also significant (β = 1.41, *p* <.001), suggesting that the effect of feedback condition was different for words in isolation and words in sentences (also see Fig. 6). Pairwise comparisons showed that for words in isolation, implicit and explicit feedback did not significantly differ from each other when averaging over feedback cycle (β = 0.06, *p* =.999). For words embedded in sentences, however, pupils slowed down more after explicit feedback than after implicit feedback (β = -1.36, *p* <.001). In addition, the interaction effect between feedback condition and feedback cycle was not significant (β = 0.22, *p* =.653). Pairwise comparisons revealed that explicit feedback did not lead to a smaller or larger slowdown than implicit feedback in both feedback cycles irrespective of word context (1: β = -0.54, *p* = 0.100; 2:β = -0.76, *p* = 0.389). See Fig. 7 for a visualization of the interaction effect.

## References

[CR1] Bai, Y., Tejedor-García, C., Hubers, F., Cucchiarini, C., & Strik, H. (2021). Automatic speech recognition technology and reading skill development in primary school. *ICERI2021 Proceed.* 10.21125/iceri.2021.1393

[CR2] Bai, Y., Hubers, F., Cucchiarini, C., & Strik, H. (2020). Asr-based evaluation and feedback for individualized reading practice. https://doi.org/10.21437/Interspeech.2020-2842

[CR3] Bai, Y., Hubers, F., Cucchiarini, C., & Strik, H. (2021). An ASR-based reading tutor for practicing reading skills in the first grade: improving performance through threshold adjustment. *Proc. IberSPEECH*. 10.21437/IberSPEECH.2021-3

[CR4] Bai, Y., Hubers, F., Cucchiarini, C., van Hout, R., & Strik, H. (2022). *The effects of implicit and explicit feedback in an asr-based reading tutor for dutch first-graders*. Incheon: Interspeech. 10.1007/s10758-025-09860-8 PMC13526040 42670340

[CR5] Bai, Y., Tejedor-García, C., Hubers, F., Cucchiarini, C., & Strik, H. (2021). An asr-based tutor for learning to read: How to optimize feedback to first graders. In A. Karpov & R. Potapova (Eds.), *Speech and computer* (pp. 58–69). Cham: Springer International Publishing.

[CR6] Balogh, J., Bernstein, J., Cheng, J., Moere, A. V., Townshend, B., & Suzuki, M. (2012). Validation of automated scoring of oral reading. *Edu. Psychol. Measur.,**72*(3), 435–452.

[CR7] Barton, K. (2022). Mumin: Multi-model inference [R package version 1.46.0]. https://CRAN.R-project.org/package=MuMIn

[CR8] Bates, D., Mächler, M., Bolker, B., & Walker, S. (2015). Fitting linear mixed-effects models using lme4. *J. Statis. Softw.,**67*(1), 1–48.

[CR9] Bernstein, J., Cheng, J., Balogh, J., & Rosenfeld, E. (2017). Studies of a Self-Administered Oral Reading Assessment. Proc. 7th ISCA Workshop on Speech and Language Technology in Education (SLaTE 2017), 172–176.

[CR10] Besser, E. D., Blackwell, L. E., & Saenz, M. (2022). Engaging students through educational podcasting: three stories of implementation. *Technol. Knowl. Learn.,**27*(3), 749–764.

[CR11] Black, M., Tepperman, J., Lee, S., Price, P., & Narayanan, S. S. (2007). Automatic detection and classification of disfluent reading miscues in young children’s speech for the purpose of assessment. *Proc. Interspeech,**2007*, 206–209.

[CR12] Bolaños, D., Cole, R. A., Ward, W., Borts, E., & Svirsky, E. (2011). Flora: fluent oral reading assessment of children’s speech. *ACM Trans. Speech Lang. Process.,**7*(4), 15.

[CR13] Butler, D. L., & Winne, P. H. (1995). Feedback and self-regulated learning: a theoretical synthesis. *Rev. Edu. Res.,**65*(3), 245–281. 10.3102/00346543065003245

[CR14] Castles, A., Rastle, K., & Nation, K. (2018). Ending the reading wars: reading acquisition from novice to expert. *Psychol. Sci. Public Interest,**19*(1), 5–51. 29890888 10.1177/1529100618772271

[CR15] Cheatham, G. A., Jimenez-Silva, M., & Park, H. (2015). Teacher feedback to support oral language learning for young dual language learners. *Early Child Develop. Care,**185*(9), 1452–1463.

[CR16] Chiang, P., & Jacobs, C. (2010). Enhancing learning using automatic speech recognition technology. *J. Edu. Technol. Syst.,**38*(1), 61–75. 10.2190/ET.38.1.e

[CR17] Clay, M. (1987). Implementing reading recovery: systemic adaptations to an educational innovation. *New Zealand J. Edu. Stud.,**22*, 35.

[CR18] Cleuren, L. (2009). Elements of speech technology based reading assessment and intervention [Doctoral dissertation, KU Leuven].

[CR19] Duchateau, J., Kong, Y., Cleuren, L., Latacz, L., Roelens, J., Samir, A., Demuynck, K., Ghesquière, P., Verhelst, W., & Van hamme, H. (2009). Developing a reading tutor: design and evaluation of dedicated speech recognition and synthesis modules. *Speech Commun.,**51*, 985–994.

[CR20] Ellis, R. (2006). Researching the effects of form-focussed instruction on l2 acquisition. *AILA Rev.,**19*(1), 18–41.

[CR21] Ellis, R. (2009). Corrective feedback and teacher development. *L2 J.,**1*(1), 10.

[CR22] Grueneberg, K., Katriel, A., Lai, J., & Feng, J. (2007). Reading companion: a interactive web-based tutor for increasing literacy skills. In C. Baranauskas, P. Palanque, J. Abascal, & S. D. J. Barbosa (Eds.), *Human-computer interaction - interact 2007* (pp. 345–348). Berlin: Springer.

[CR23] Hagen, A., Pellom, B., & Cole, R. (2003). Children’s speech recognition with application to interactive books and tutors. 2003 IEEE Workshop on Automatic Speech Recognition and Understanding (IEEE Cat. No.03EX721), 186–191.

[CR24] Hattie, J., & Timperley, H. (2007). The power of feedback. *Rev. Edu. Res.,**77*(1), 81–112. 10.3102/003465430298487

[CR25] Heck, R. H., Thomas, S. L., & Tabata, L. N. (2014). Multilevel and longitudinal modeling with ibm spss (2nd ed.) Routledge/Taylor & Francis Group. 10.4324/9780203701249

[CR26] Heubusch, J., & Lloyd, J. (1998). Corrective feedback in oral reading. *J. Behav. Edu.,**08*, 63–79.

[CR27] Kantor, A., Cerňak, M., Havelka, J., Huber, S., Kleindienst, J., & Gonzalez, D. B. (2012). Reading companion: the technical and social design of an automated reading tutor. Proc. 3rd Workshop on Child, Computer and Interaction (WOCCI 2012), 53–59.

[CR28] Kendeou, P., Van den Broek, P., White, M. J., & Lynch, J. S. (2009). Predicting reading comprehension in early elementary school: the independent contributions of oral language and decoding skills. *J. Edu. Psychol.,**101*(4), 765.

[CR29] Kuznetsova, A., Brockhoff, P. B., & Christensen, R. H. B. (2017). lmerTest package: tests in linear mixed effects models. *J. Statis. Softw.,**82*(13), 1–26.

[CR30] Li, X., Deng, L., Ju, Y.-C., & Acero, A. (2008). Automatic children’s reading tutor on hand-held devices. *Proc. Interspeech,**2008*, 1733–1736.

[CR31] Lippert, A., Shubeck, K., Morgan, B., Hampton, A., & Graesser, A. (2020). Multiple agent designs in conversational intelligent tutoring systems. *Technol. Knowl. Learn.,**25*(3), 443–463.

[CR32] Lonigan, C. J., Schatschneider, C., Westberg, L., et al. (2008). *Identification of children’s skills and abilities linked to later outcomes in reading, writing, and spelling* (pp. 55–106). Developing early literacy: Report of the national early literacy panel.

[CR33] Loukina, A., Klebanov, B. B., Lange, P., Qian, Y., Gyawali, B., Madnani, N., Misra, A., Zechner, K., Wang, Z., & Sabatini, J. (2019). Automated estimation of oral reading fluency during summer camp e-book reading with MyTurnToRead. *Proc. Interspeech,**2019*, 21–25.

[CR34] Loukina, A., Klebanov, B. B., Lange, P. L., Gyawali, B., & Qian, Y. (2017). Developing speech processing technologies for shared book reading with a computer. WOCCI, 46–51.

[CR35] Lüdecke, D. (2022). Sjplot: Data visualization for statistics in social science [R package version 2.8.11]. https://CRAN.R-project.org/package=sjPlot

[CR36] Lyster, R. (2004). Differential effects of prompts and recasts in form-focused instruction. *Stud. Second Lang. Acquis.,**26*(3), 399–432.

[CR37] Madnani, N., Beigman Klebanov, B., Loukina, A., Gyawali, B., Lange, P., Sabatini, J., & Flor, M. (2019). My turn to read: An interleaved E-book reading tool for developing and struggling readers. Proceedings of the 57th Annual Meeting of the Association for Computational Linguistics: System Demonstrations, 141–146.

[CR38] Martin-Chang, S. (2017). Learning to read with and without feedback, in and out of context. *J. Edu. Psychol.,**109*(2), 233.

[CR39] McCandliss, B. D., Beck, I. L., Sandak, R., & Perfetti, C. A. (2003). Focusing attention on decoding for children with poor reading skills: Design and preliminary tests of the word building intervention. *Sci. Stud. Read.,**7*(1), 75–104. 10.1207/S1532799XSSR0701_05

[CR40] Meredith, T. R. (2015). Using augmented reality tools to enhance children’s library services. *Technol. Knowl. Learn.,**20*(1), 71–77.

[CR41] Metcalfe, J., Kornell, N., & Finn, B. (2009). Delayed versus immediate feedback in children’s and adults’ vocabulary learning. *Mem. Cogn.,**37*(8), 1077–1087. 10.3758/MC.37.8.1077 19933453

[CR42] Mommers, M., Verhoeven, L., & Van der Linden, S. (1990). *Veilig Leren Lezen*. Zwijsen.

[CR43] Mostow, J. (2016). Project listen’s reading tutor.

[CR44] Mostow, J., & Aist, G. (1999). Giving help and praise in a reading tutor with imperfect listening - because automated speech recognition means never being able to say you’re certain. *CALICO J.,**16*(3), 407–424.

[CR45] Mostow, J., Aist, G., Burkhead, P., Corbett, A., Cuneo, A., Eitelman, S., Huang, C., Junker, B., Sklar, M. B., & Tobin, B. (2003). Evaluation of an automated reading tutor that listens: comparison to human tutoring and classroom instruction. *J. Edu. Comput. Res.,**29*(1), 61–117.

[CR46] Mostow, J., & Aist, G. (2001). Evaluating tutors that listen: an overview of project listen. *Int. J. Speech Technol.,**4*(3), 285–296. 10.1023/A:1011334225888

[CR47] Mostow, J., Nelson-Taylor, J., & Beck, J. E. (2013). Computer-guided oral reading versus independent practice: comparison of sustained silent reading to an automated reading tutor that listens. *J. Edu. Comput. Res.,**49*(2), 249–276.

[CR48] National Reading Panel. (2000). Teaching children to read: An evidence-based assessment of the scientific research literature on reading and its implications for reading instruction. https://www.nichd.nih.gov/publications/pubs/nrp/Documents/report.pdf

[CR49] Netz, H., & Fogel, O. (2019). Input-providing vs. output-pushing corrective feedback in dyadic tutoring sessions. *System,**87*, 102159.

[CR50] Nicol, D. J., & Macfarlane-Dick, D. (2006). Formative assessment and self-regulated learning: a model and seven principles of good feedback practice. *Stud. Higher Edu.,**31*(2), 199–218. 10.1080/03075070600572090

[CR51] Pany, D., & McCoy, K. M. (1988). Effects of corrective feedback on word accuracy and reading comprehension of readers with learning disabilities. *J. Learn. Disab.,**21*(9), 546–550. 10.1177/002221948802100905 3199026

[CR52] Perkins, V. L. (1988). Feedback effects on oral reading errors of children with learning disabilities. *J. Learn. Disab.,**21*(4), 244–248. 10.1177/002221948802100412 3373125

[CR53] R Core Team. (2022). R: A language and environment for statistical computing. R Foundation for Statistical Computing. Vienna, Austria. https://www.R-project.org/

[CR54] Reeder, K., Shapiro, J., Wakefield, J., & D’Silva, R. (2015). Speech recognition software contributes to reading development for young learners of english. *Int. J. Comput. Assist. Lang. Learn. Teach. (IJCALLT),**5*(3), 60–74.

[CR55] Spaai, G. W., Ellermann, H. H., & Reitsma, P. (1991). Effects of segmented and whole-word sound feedback on learning to read single words. *J. Edu. Res.,**84*(4), 204–214.

[CR56] Speece, D. L., & Ritchey, K. D. (2005). A longitudinal study of the development of oral reading fluency in young children at risk for reading failure. *J. Learn. Disab.,**38*(5), 387–399. 10.1177/00222194050380050201 16329440

[CR57] Swart, E. K., Nielen, T. M., & Sikkema - de Jong, M. T. (2019). Supporting learning from text: A meta-analysis on the timing and content of effective feedback. *Edu. Res. Rev.,**28*, 100296.

[CR58] Van der Kleij, F. M., Feskens, R. C., & Eggen, T. J. H. M. (2015). Effects of feedback in a computer-based learning environment on students’ learning outcomes: a meta-analysis. *Rev. Edu. Res.,**85*(4), 475–511. 10.3102/0034654314564881

[CR59] Van Rossum, G., & Drake, F. L. (2009). *Python 3 reference manual*. CreateSpace.

[CR60] Verhoeven, L., & Leeuwe, J. (2009). Modeling the growth of word-decoding skills: evidence from dutch. *Sci. Stud. Read.,**13*, 205–223.

[CR61] Watson, M., Fore, C., III., & Boon, R. T. (2009). Corrective feedback of oral decoding errors for diverse learners with reading disabilities: the effects of two methods on reading fluency. *Int. J. Special Edu.,**24*(1), 20–31.

[CR62] Wickham, H., Averick, M., Bryan, J., Chang, W., McGowan, L. D., François, R., Grolemund, G., Hayes, A., Henry, L., Hester, J., Kuhn, M., Pedersen, T. L., Miller, E., Bache, S. M., Müller, K., Ooms, J., Robinson, D., Seidel, D. P., Spinu, V., Yutani, H., et al. (2019). Welcome to the tidyverse. *J. Open Source Softw.,**4*(43), 1686.

